# Analysis of Septin Reorganization at Cytokinesis Using Polarized Fluorescence Microscopy

**DOI:** 10.3389/fcell.2017.00042

**Published:** 2017-05-03

**Authors:** Molly McQuilken, Maximilian S. Jentzsch, Amitabh Verma, Shalin B. Mehta, Rudolf Oldenbourg, Amy S. Gladfelter

**Affiliations:** ^1^Department of Biology, University of North Carolina at Chapel HillChapel Hill, NC, USA; ^2^Department of Biological Sciences, Dartmouth CollegeHanover, NH, USA; ^3^Marine Biological Laboratory, Bell Center for Regenerative MedicineWoods Hole, MA, USA; ^4^Department of Physics, Brown UniversityProvidence, RI, USA

**Keywords:** septins, cytokinesis, polarized light microscopy, Shs1

## Abstract

Septins are conserved filament-forming proteins that act in diverse cellular processes. They closely associate with membranes and, in some systems, components of the cytoskeleton. It is not well understood how filaments assemble into higher-order structures *in vivo* or how they are remodeled throughout the cell cycle. In the budding yeast *S. cerevisiae*, septins are found through most of the cell cycle in an hourglass organization at the mother-bud neck until cytokinesis when the collar splits into two rings that disassemble prior to the next cell cycle. Experiments using polarized fluorescence microscopy have suggested that septins are arranged in ordered, paired filaments in the hourglass and undergo a coordinated 90° reorientation during splitting at cytokinesis. This apparent reorganization could be due to two orthogonal populations of filaments disassembling and reassembling or being preferentially retained at cytokinesis. In support of this idea, we report a decrease in septin concentration at the mother-bud neck during cytokinesis consistent with other reports and the timing of the decrease depends on known septin regulators including the Gin4 kinase. We took a candidate-based approach to examine what factors control reorientation during splitting and used polarized fluorescence microscopy to screen mutant yeast strains deficient in septin interacting proteins. Using this method, we have linked known septin regulators to different aspects of the assembly, stability, and reorganization of septin assemblies. The data support that ring splitting requires Gin4 activity and an anillin-like protein Bud4, and normal accumulation of septins at the ring requires phosphorylation of Shs1. We found distinct regulatory requirements for septin organization in the hourglass compared to split rings. We propose that septin subpopulations can vary in their localization and assembly/disassembly behavior in a cell-cycle dependent manner at cytokinesis.

## Introduction

Septins are a conserved family of cytoskeletal GTP-binding proteins that function in many cellular processes including cytokinesis, cell polarity, and membrane remodeling in many eukaryotic cell types (Gilden and Krummel, 2010; Mostowy and Cossart, 2012; Khan et al., 2015). To contribute to these diverse processes, septins polymerize into filaments and then assemble into higher-order structures associated with the cell cortex (Frazier et al., 1998; Bertin et al., 2008; Spiliotis and Gladfelter, 2012; Bridges et al., 2014). In the budding yeast *Saccharomyces cerevisiae*, septins assemble into a ring at the new bud site and this ring transitions into an hourglass which splits into two rings at cytokinesis (Haarer and Pringle, 1987; Ford and Pringle, 1991; Kim et al., 1991). The formation of higher-order septin structures is required for proper septin function, and for decades the underlying architecture of these higher-order structures remained uncertain (Byers and Goetsch, 1976; Rodal et al., 2005; Bertin et al., 2012). Recent platinum-replica transmission electron microscopy studies provided clear ultrastructural details of septin higher-order structures in yeast over the course of the cell cycle (Ong et al., 2014). In this approach, predominantly paired filaments parallel to the mother-bud axis were detectable in the hourglass early in the cell cycle, whereas at cytokinesis two orthogonal arrays (parallel and circumferential to axis) of paired septin filaments could be seen and finally after splitting all filaments appeared to be circumferential. The exact ultrastructure in a mature hourglass prior to cytokinesis is not clear and it is possible that the circumferential filaments assemble prior to the onset of cytokinesis as the intensity of septins continues to increase through time in G2/M (Chen et al., 2011). It is not yet clear what the function is of the process of splitting the hourglass into two rings. There is evidence that the split structure forms a corral for the cytokinetic apparatus to trap it locally however it is not clear that the corral is needed for efficient cytokinesis (Dobbelaere and Barral, 2004; Wloka et al., 2011).

The budding yeast *S. cerevisiae*, where septins were first discovered, contains 5 mitotic septins: three septins that assembly into the core of the heteromeric rod (Cdc12, Cdc10, Cdc3) while either Cdc11 or Shs1 occupies the terminal position (Hartwell, 1971; Bertin et al., 2008; Garcia et al., 2011). *In vivo* it is not yet definitive the proportion of rods that have Shs1 or Cdc11 at the terminal positions or if there are rods that are polar and have Cdc11 on one end and Shs1 on the other end. The molecular role of Shs1 is unclear, since it is clearly necessary for higher order structure organization but it is not required for cells to undergo cytokinesis except in sensitized backgrounds (Hartwell, 1971; Mino et al., 1998; Garcia et al., 2011; Ong et al., 2014; Booth et al., 2015). Previous studies have assessed the role of C-terminal extension (CTE) in Shs1 localization and function in both *S. cerevisiae* and *Ashbya gossypii* (Versele and Thorner, 2004; Meseroll et al., 2012, 2013; Finnigan et al., 2015). In *A. gossypii*, the CTE is required to build structures of a specific size and the CTE is phosphorylated on multiple sites *in vivo*. The phosphorylation is not essential for ring scaling but it does contribute to protein turnover in higher-order assemblies and phosphomimetic alleles are lethal (Meseroll et al., 2012). Similarly, the CTE of ScShs1 is highly phosphorylated and the sites are not essential for viability in a sensitized background (Egelhofer et al., 2008; Garcia et al., 2011). However, whether or not the sites contribute to reorganization or splitting of the ring at cytokinesis has not been investigated. A hypothesis we test in this paper is if regulation of Shs1 is required for septin reorganization at cytokinesis.

Insights into septin organization through the cell cycle in live cells have come from using polarized fluorescence microscopy. This microscopy technique provides organizational information in addition to localization of cellular structures through fluorescence imaging. The approach takes advantage of the existence of the dipole moment of GFP, which allows GFP to be preferentially excited by and emit linearly polarized light parallel to the dipole moment (Inoué et al., 2002). If the movement of GFP is restricted relative to its septin fusion partner, then the dipole moment of GFP will act as a readout of underlying septin organization (Vrabioiu and Mitchison, 2006, 2007). Previously, polarized fluorescence microscopy was used by our group and others to assess the organization of septin higher order structures over the course of the cell cycle and found septins to be highly anisotropic (ordered). It was shown that septins rapidly and dramatically reorganize their ensemble organization by 90° over the course of septin ring splitting (Vrabioiu and Mitchison, 2006, 2007; DeMay et al., 2011a,b).

We set out to understand the root of this striking rearrangement of the highly-ordered septin cortex during cytokinesis. The molecular basis for a large-scale rearrangement of a network of cytoskeletal filaments between two, highly anisotropic yet orthogonal states is difficult to envision. Several models for this transition have been proposed previously: (1) a concerted rotation of the entire higher-order assembly; (2) the selective departure of a majority population of filaments that then “reveals” a second, orthogonal population; and (3) a triggered disassembly and reassembly of filaments into a new ensemble that is perpendicular to the starting state (Vrabioiu and Mitchison, 2006; DeMay et al., 2011a). Work from the Barral lab showed that the collar becomes more “fluid” at cytokinesis based on recovery from photobleaching which could be consistent with either a rotation or disassembly/reassembly model (Dobbelaere and Barral, 2004). Analysis of septin filaments *in vitro* have shown them to be highly flexible, that they associate with the membrane through avidity and that longer filaments are more stably bound (Bridges et al., 2014, 2016). These properties could enable a regulated fragmentation and departure of a population of filaments, key components of models 2 and 3. Strong support for the third model came from EM ultrastructural and photoactivation studies from Bi and Svitkina, making it the most well substantiated at this point, although it is still possible that circumferential rings assemble in mature hourglasses and these could be retained at cytokinesis (Ong et al., 2014). The key question is: what promotes such a swift rearrangement of molecular order within such a small window of time and space?

Our goal in this study was to begin to identify the molecular requirements of septin reorganization at cytokinesis. We assessed the organization of septins in a panel of mutant yeast strains using polarized fluorescence microscopy. Our results suggest phosphorylation-based modifications of septins, Gin4 kinase activity and the anillin-like protein Bud4 are important for maintaining a subpopulation of septins at the bud neck before, during and after cytokinesis.

## Materials and methods

### Yeast strains and strain construction

We generated *S. cerevisiae* Cdc12-conGFP (AGY169; DHD5 yeast with AGB467: pRS416-Sc*CDC12*-conGFP3) based on rationale from previous work (Vrabioiu and Mitchison, 2006, 2007; DeMay et al., 2011a,b). pRS416-Sc*CDC12*-conGFP3 was generated by removing nucleotides encoding 3 amino acids of the N-terminal alpha-helix of GFP and 4 amino acids of the C-terminal region of Cdc12. AGB455 (pSc*CDC12*) was generated by amplifying Sc*CDC12* from *S. cerevisiae* genomic DNA using the oligonucleotides AGO1181 and AGO1182. The PCR product was ligated into the pRS416 plasmid (AGB441) and verified by test digestions using PspXI and SlaI and sequencing (Dartmouth College Core Facility, Hanover, NH). Next, the plasmid AGB459 (pScCdc12-GFP) was generated by first amplifying the GFP insert from the plasmid AGB005 using the oligonucleotides AGO1187 and AGO1188 and ligating the product into the digested AGB455 plasmid. Again this plasmid was verified with test digestions and sequencing (Dartmouth College Core Facility, Hanover, NH). Constraining of GFP to Cdc12 was completed using PCR to amplify the entire AGB459 using the oligonucleotides AGO1203 and AGO1204 which contain homology over the *CDC12*-GFP linker and this amplification removed nucleotides encoding 4 amino acids from the N-terminus of GFP and 3 amino acids from the C-terminus of Cdc12. AGB467 (pRS416-Sc*CDC12*-conGFP) was verified by sequencing (Dartmouth College Core Facility, Hanover, NH). Once verified, AGB467 was transformed into wild-type lab strain (AGY000), and the following yeast deletion strains: *bnr1*Δ*, bni1*Δ*, bud4*Δ*, cla4*Δ*, gin4*Δ*, rts1*Δ, and *shs1*Δ (kindly provided by Erfei Bi, AGY028-AGY035 to generate AGY066-AGY073). Doug Kellogg generously supplied us with *shs1* phosphomutant strains (Egelhofer et al., 2008) and the plasmid AGB467 was transformed into these strains as well to generate AGY317-AGY320. To generate strains to measure septin intensity over the cell cycle the plasmid AGB553 (E1915 YIplac128-GFP-Sc*CDC3*:*LEU* a gift from Erfei Bi) was integrated into the same strains as the AGB467 plasmid generating the strains AGY131-AGY137, and AGY323-AGY327. All strain, plasmid, and oligonucleotide information is present in Tables 1–3.

**Table 1:** **Yeast strains used in this study**.

**Strain**	**Relevant Genotype**	**Reference**
AGY000	DHD5 (MATa/MATα, ura3-52/ura3-52, leu2-3,112/leu2-3, 112, his3-11, 25/his3-11,15)	
AGY028	YEF3572 Mat a *bud4Δ*:his	Erfei Bi
AGY029	YEF3922 *rts1Δ*:kan	Erfei Bi
AGY030	YEF1342 Mata *cla4Δ*:his	Erfei Bi
AGY031	YEF1238 Mat a *gin4Δ*:trp	Erfei Bi
AGY032	YEF5687 Mat a *shs1Δ*:trp	Erfei Bi
AGY034	YEF6005 Mat α *bni1Δ*:his	Erfei Bi
AGY035	YEF1732 Mat α *bnr1Δ*:his	Erfei Bi
AGY066	pRS416-Sc*CDC12*-conGFP/YEF3572 Mat a *bud4Δ*:his	This study
AGY067	pRS416-Sc*CDC12*-conGFP/YEF3922 *rts1Δ*:kan	This study
AGY068	pRS416-Sc*CDC12*-conGFP/YEF1342 Mata *cla4Δ*:his	This study
AGY069	pRS416-Sc*CDC12*-conGFP/YEF1238 Mat a *gin4Δ*:trp	This study
AGY070	pRS416-Sc*CDC12*-conGFP/YEF5687 Mat a *shs1Δ*:trp	This study
AGY072	pRS416-Sc*CDC12*-conGFP/YEF6005 Mat α *bni1Δ*:his	This study
AGY073	pRS416-Sc*CDC12*-conGFP/YEF1732 Mat α *bnr1Δ*:his	This study
AGY075	Sc*CDC11*-GFP::His, Sc*SHS1*-mCherry::Gen	H. Ewers
AGY131	E1915 YIplac128-GFP-Sc*CDC3*:LEU/YEF1238 Mat a *gin4Δ*:trp	This study
AGY132	E1915 YIplac128-GFP-Sc*CDC3*:LEU/YEF5687 Mat a *shs1Δ*:trp	This study
AGY133	E1915 YIplac128-GFP-Sc*CDC3*:LEU/YEF3572 Mat a *bud4Δ*:his	This study
AGY134	E1915 YIplac128-GFP-Sc*CDC3*:LEU/YEF6005 Mat α *bni1Δ*:his	This study
AGY135	E1915 YIplac128-GFP-Sc*CDC3*:LEU/YEF1732 Mat α *bnr1Δ*:his	This study
AGY136	E1915 YIplac128-GFP-Sc*CDC3*:LEU/YEF3922 *rts1Δ*:kan	This study
AGY137	E1915 YIplac128-GFP-Sc*CDC3*:LEU/YEF1342 Mata *cla4Δ*:his	This study
AGY169	pRS416-Sc*CDC12*-conGFP/DHD5	This study
AGY311	DK186 (Mat a, his3-11,15, leu2-3, 112, trp1-1, ura3-52, ade2-1, can1-100, GAL+, bar1)	Egelhofer et al., 2008
AGY313	DK912 (Mat a, his3-11,15, leu2-3, 112, trp1-1, ura3-52, ade2-1, can1-100, GAL+, bar1, *shs1Δ*::shs1-ps2)	Egelhofer et al., 2008
AGY314	DK966 (Mat a, his3-11,15, leu2-3, 112, trp1-1, ura3-52, ade2-1, can1-100, GAL+, bar1, *shs1Δ*::shs1-ps1)	Egelhofer et al., 2008
AGY315	DK985 (Mat a, his3-11,15, leu2-3, 112, trp1-1, ura3-52, ade2-1, can1-100, GAL+, bar1, *shs1Δ*::shs1-ps4)	Egelhofer et al., 2008
AGY317	pRS416-Sc*CDC12*-conGFP/DK186	This study
AGY318	pRS416-Sc*CDC12*-conGFP/DK912	This study
AGY319	pRS416-Sc*CDC12*-conGFP/DK966	This study
AGY320	pRS416-Sc*CDC12*-conGFP/DK985	This study
AGY323	E1915 YIplac128-GFP-Sc*CDC3*:LEU/DK186	This study
AGY325	E1915 YIplac128-GFP-Sc*CDC3*:LEU/DK912	This study
AGY326	E1915 YIplac128-GFP-Sc*CDC3*:LEU/DK966	This study
AGY327	E1915 YIplac128-GFP-Sc*CDC3*:LEU/DK985	This study
DLY5487	*SHS1*-GFP::kan-1, mat a	Danny Lew

**Table 2:** **Plasmids used in this study**.

**Plasmid #**	**Name**	**Vector**	**Relevant insert**	**Reference**
AGB005	pAGT141	pUC19	GFP	
AGB441	pRS416	pRS416	–	
AGB455	pRS416-Sc*CDC12* locus-stop	pRS416	Sc*CDC12* locus-stop	This study
AGB459	pRS416-Sc*CDC12*-GFP	pRS416	GFP	This Study
AGB467	pRS416-Sc*CDC12*-conGFP	pRS416	conGFP4-GEN3 (4D4)	This study
AGB553	E1915 YIplac128-GFP-Sc*CDC3*:LEU	YIplac128	GFP-Sc*CDC3*	Erfei Bi

**Table 3:** **Oligonucleotides used in this study**.

**Plasmid #**	**Name**	**Sequence 5′-3′**
AGO1181	ScCdc12 locus PspXI F	GGTGCCTCGAGGGGCTTCAAAACTGCTAGGTCGGATTC
AGO1182	ScCdc12 locus SalI R	GGAGGTCGACTTTTAAATGGGATTTTTTTACTTGCAAGCTTTTGACCTGCTCTTC
AGO1187	Sc GFP tagging SalI F	GGTGGTCGACGGCGCGGGCGCAGGTGCCGGTGCAAGTAAAGGAGAAGAACTTTTCACTGGAGTTGTCCC
AGO1188	Sc GFP tagging EcoRI R	GGCGGAATTCCTATGCGTCCATCTTTACAGTCC
AGO1203	MVB128 Sc*Cdc12*-conGFP F	GCTTGCAAGTAAAAAAATCCGAACTTTTCACTGGAGTTG
AGO1204	MVB128 Sc*Cdc12*-conGFP R	CAACTCCAGTGAAAAGTTCGGATTTTTTTACTTGCAAGC

### Yeast culture and preparation

For imaging, *S. cerevisiae* cells were grown overnight in appropriate media with proper selection for the specific plasmids, collected by centrifugation, and resuspended in 2x low fluorescence minimal media (LFMM). Cells were mounted on gel pads of 1.4% agarose and LFMM on glass depression slide, covered with a coverslip (no. 1.5), sealed with Valap, and imaged.

### Time-lapse imaging and intensity analysis

Time-lapse recordings for estimating septin intensity of mutant strains through the cell cycle were acquired with a Nikon Eclipse Ti-E inverted wide-field microscope equipped with a 60x (1.4 N.A.) plan-apochromat oil objective and a Andor Zyla 4.2 sCMOS camera. A Chroma DAPI/FITC/TRITC/Cy5 quad filter set was used for fluorescent imaging of GFP. The fluorescent light source was a Spectra LED lamphead and images were acquired with 12% laser power, 100 ms exposure, and 90 s time intervals.

Using FIJI, individual cells were cropped from the timelapse recordings and the mean background-subtracted septin intensity at the mother-bud neck were determined for every time point for a given cell using MATLAB. These septin intensity values were saved and plotted in an Excel file. For every cell, a time zero (***t0***) was manually determined. This ***t0*** represented the maximum septin intensity value close (not more than 4–5 time points) to the visible ring split. In most cases the quantitatively determined ***t0*** was not more than two time points (3 min) away from the visible split. This quantitative approach was taken to account for different visible split times depending on how a given image was automatically contrasted by FIJI. ***t0*** therefore represented the quantitative onset of ring splitting. Since the primary focus of this study was the septin concentration change during ring splitting, all following analyses were limited to 24 min before and after ***t0***. Percent change over the entire 24 min was determined and reported in Table 4. Since the majority of the strains analyzed showed biphasic septin disassembly rates over the course of ring splitting, the disassembly rate was determined for both phases of disassembly (phase 1 and phase 2). Phase 1 and phase 2 were distinguished by the different slopes of the biphasic intensity curves. If a strain did not show biphasic disassembly, then it is reported as monophasic and the intensity from *t* = 0 to *t* = 24 min after splitting; Phase 2 is the rate of disassembly from the last time point of Phase 1 to *t* = 24. After disassembly rates were determined, the fold change compared to wild-type was calculated.

**Table 4:** **Average percent disassembly, rate change, rate fold change for each phase of disassembly observed**.

**Strain**	**% disassembly (phase1)**	**Rate (% disassembly/min) phase 1**	**Rate (% disassembly/min) phase 2**	**Monophasic (disassembly/min)**	**Rate fold change phase 1**	**Rate fold change phase 2**
*CDC3*-GFP	56.6	6.3	3.4	–	1.0	1.0
*CDC11*-GFP	58.9	6.5	3.4	–	1.0	1.0
*SHS1*-GFP	51.0	5.7	2.5	–	0.9	0.7
*SHS1*-GFP (DLY)	52.4	7.0	2.3	–	1.1	0.7
*bni1Δ*	62.5	10.4	1.7	–	1.7	0.5
*bnr1Δ*	74.7	10.0	3.3	–	1.6	1.0
bud4Δ^*^	98.3	–	–	12.0	1.9	3.5
*cla4Δ*	41.2	6.9	4.7	–	1.1	1.4
*gin4Δ*	51.3	–	–	2.1	0.3	0.6
*rts1Δ*	49.2	8.2	4.1	–	1.3	1.2
*shs1Δ*	56.7	–	–	2.4	0.4	0.7
*shs1ΔC*	99.5	10.3	5.9	–	1.6	1.8
*CDC3*-GFP (W303)	99.1	–	–	4.1	1.0	–
*shs1Δ-ps1*	92.8	–	–	3.9	0.9	–
*shs1Δ-ps2*	97.7	–	–	4.1	1.0	–
*shs1Δ-ps4*	100.0	–	–	4.0	1.0	–

*Rate fold change is normalized to the relevant wild-type background, Phase 1 was identified visually by the first rate of disassembly in the slopes of the biphasic intensity curves. If a strain did not show biphasic disassembly, then it is reported as monophasic and the intensity from t = 0 to t = 24 min after splitting; Phase 2 is the rate of disassembly from the last time point of Phase 1 to t = 24*.

* *bud4Δ decreased in intensity faster than wild-type yeast; therefore, disassembly rate was determined only for the first 7.5 min*.

### Polarization microscopy

All polarization fluorescence images (except the *shs1*-phosphomutant strains) were acquired using the MF-PolScope (Abrahamsson et al., 2015). The MF-PolScope was set up on an inverted Olympus IX-83 microscope body, equipped with an Olympus 60x (1.3 N.A.) silicon-oil immersion objective, and the LC-compensator for polarization-controlled excitation light, with a green 515/30 nm emission filter (Semrock) placed before an EMCCD camera (Andor iXon-888). The fluorescence light source used was a blue LED module of an X-cite XLED1 (Lumen Dynamics). Images were acquired through Micro-manager (www.micro-manager.org) using the OpenPolScope software (www.openpolscope.org).

Polarized fluorescence measurements of the *shs1*-phosphomutant strains and *bud4*Δ splitting events were acquired with the Zeiss Axio Imager.M1 wide-field microscope with five linear polarized filters (Chroma 21033a) in a filter wheel (Ludl Electronic Products, cat. no. 99A075) and UV blocker 420 nm LP emission filter 32 nm (Chroma, cat. no. E420LPv2-32). The fluorescent light source used was an EXFO X-Cite 120 lamp. Images were acquired through Micro-manager (www.micro-manager.org) using the OpenPolScope software (www.openpolscope.org).

Once acquired, all polarization images were analyzed using the OpenPolScope software. Analysis involved internal background corrections of every cell, performed by selecting a ROI in the cytoplasm, and a ROI in the septin hourglass structure and then processed for anisotropy. The anisotropy formula calculates $\frac{I_{max}-I_{min}}{I_{max}+I_{min}}$ in each pixel, based on the bleach corrected fluorescence intensity values. Circular statistics were used to calculate the ensemble orientation and anisotropy for each given septin structure. To do this, a 6 × 6 pixel ROI was selected in the center of the septin structure so as to exclude edges of the septin structure with isotropic measures from GFP β-barrel orientation along the curve of the septin ring. The pixel-by-pixel orientation, anisotropy, and intensity for each septin structure was then exported, and used to calculate the ensemble orientation weighted by anisotropy and intensity of each ring, and of the entire population of cells. In addition we also calculated the population variance ($PopVar=1-\left(\frac{Vector\;Average\;Anisotropy*Intensity}{Scalar\;Anisotropy*Intensity}\right)$). A detailed description of how to use the OpenPolScope software for polarization imaging and analysis is present in *Current Protocols in Cell Biology* (McQuilken et al., 2015).

## Results

### Septin concentration at the bud neck decreases at ring splitting for multiple septin subunits

Previous data from our lab and others have shown that there is a 90° reorientation of the organization within the septin hourglass structure over the course of septin ring splitting at cytokinesis in *S. cerevisiae* cells (Vrabioiu and Mitchison, 2006; DeMay et al., 2011a,b). We had hypothesized in our work that one explanation of the apparent reorganization is that there are two populations of septins in the hourglass and this could be seen by Ong et al. (2014) at least at the onset of cytokinesis. Upon ring splitting, one of these sub-populations is thought to leave the bud neck and another population assembles and/or is retained perpendicular to the original population. What drives the disassembly and reassembly process of septins within such a small region of the cortex in a short window of the cell cycle?

**Figure 1:**
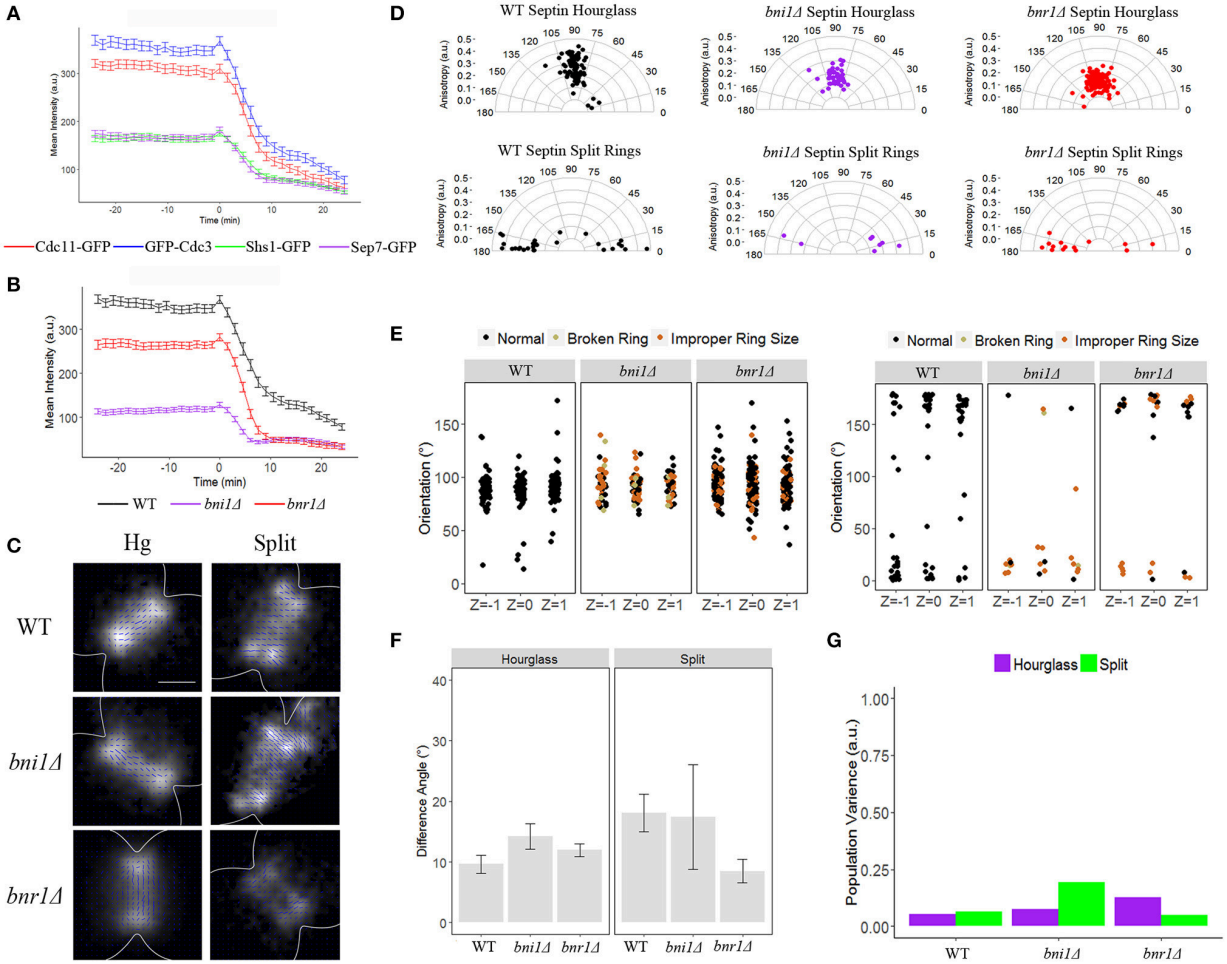
**The formins, Bni1 and Bnr1, are not required for the reorganization of the septin ring. (A)** Septin intensity of Cdc11-GFP (red), GFP-Cdc3 (blue), Shs1-GFP (green), and Sep7-GFP (purple) over the course of septin ring splitting (time = 0). Error bars represent standard error for each time point (*N* = 100 cells). **(B)** Septin intensity of GFP-Cdc3 in wild-type (black), *bni1*Δ (purple), and *bnr1*Δ (red) over the course of septin ring splitting (time = 0). Error bars represent standard error for each time point (*N* = 100 cells). **(C)** Representative polarization images of wild-type, *bni1*Δ and *bnr1*Δ cells expressing ScCdc12-conGFP in the hourglass and split structures. The blue lines represent the calculated dipole orientation and their length is scaled according to anisotropy. Scale bar 0.5 μm. **(D)** Polar plots of net dipole orientations for individual hourglass and split septin rings scaled by anisotropy (hourglass *N* > 45 cells, split rings *N* > 8 cells). **(E)** Scatter plot of net dipole orientations for individual hourglass and split septin rings in three different focal planes. Color scale indicates visible phenotypes of septin rings [broken ring (tan), improper ring size (orange), (hourglass *N* > 30 cells, split rings *N* > 8 cells)]. **(F)** Difference in net orientation measures between top and bottom focal planes for hourglass and split structures. Error bars denote standard error. **(G)** Population variance in hourglass and split ring structures.

We hypothesized that whether a given septin filament is released or stays localized at the neck could be explained by the composition of septin heteromeric rods, specifically the terminal subunit, which is occupied by either Cdc11 and/or Shs1. If Cdc11 or Shs1 specified which complexes were retained or exited from the neck, we predicted that Cdc11 and Shs1 would display different rates of change in concentration at splitting. To assess this, we monitored the abundance of 3 different septins at the mother-bud neck over the course of septin ring splitting. We used widefield fluorescence microscopy to image GFP-Cdc3, Shs1-GFP (in two different strain backgrounds), and Cdc11-GFP every 90 s to ensure sufficient time resolution for assessing intensity differences and kinetics (see *Materials and Methods*). Using two different Shs1-GFP strains we observed the accumulation of Shs1 in the septin hourglass is only ~50% of the level of either Cdc3 or Cdc11 (Figure 1A). Interestingly, we also observed that the decrease of all three septins over the course of ring splitting occurred in a biphasic manner. The first phase occurred within the first ~10 min after ring splitting. This first phase of septins leaving the bud neck is consistent with the timing of the isotropic “transition” period observed previously (DeMay et al., 2011a). Time-lapse imaging indicated that all septin proteins decrease in abundance with comparable rates during ring splitting and decrease by ~50% in concentration over the first phase of disassembly (phase 1), regardless of their starting abundance (Figure 1A, Table 4, and consistent with what was seen for Cdc3 in Dobbelaere et al., 2003; Wloka et al., 2011). When we measure the difference in disassembly rates over the first phase of disassembly between the different septins there was little difference in the rates (Table 4). Additionally, when Cdc11 and Shs1 are tagged in the same cell with different fluorophores, the decrease in abundance at the neck is nearly simultaneous for each protein, further indicating that these two subunits behave similarly with regard to septin dynamics in this part of the cell cycle (discrepancy between times of −0.3 ± 0.3 min, *N* = 40 cells, difference not significant from 0 with *p* > 0.41). These data indicate that the difference between the septin populations that stay associated with the cortex and that are released into the cytoplasm is not simply the presence or absence of Cdc11 or Shs1.

### Septin reorganization does not require the formins

We next used time-lapse imaging and polarization microscopy to assess the contribution of septin interacting proteins on the organization and behavior of septins in the splitting transition. We first tested the hypothesis that a subset of septins could be specified to disassemble or be anchored via connections with F-actin assemblies. The yeast formin Bnr1 is regulated by Shs1 indicating these two factors are functionally interacting at this window of the cell cycle (Buttery et al., 2012). We assessed how the two formins, Bni1 and Bnr1, influence septin intensity changes and reorganization over septin ring splitting (Figure 1B). In strains lacking formins we observed a decrease in the initial intensity of septins in the hourglass, suggesting the actin cytoskeleton contributes partially to the accumulation of septins at the mother bud neck prior to ring splitting. This defect was most severe in *bni1*Δ, where septin intensity at the hourglass was reduced by ~65% relative to the hourglass in wild-type cells. However, the rate at which the septin sub-population exited the bud-neck during ring splitting was again observed as a biphasic disassembly, was not substantially affected in any of the strains (0.7-fold faster for phase 1 and 0.5-fold slower for phase 2 in *bni1*Δ and 0.6-fold faster in phase 1 and no fold change difference for phase 2 *bnr1*Δ, Table 4) consistent with previous reports (Feng et al., 2015).

We next asked if the formins were required for the characteristic 90° reorganization. To address this, we used polarization microscopy to measure the organization of the septin hourglass and split rings in wild-type, *bni1*Δ, and *bnr1*Δ strains containing Cdc12-conGFP (see *Materials and Methods*, Figures 1C–E). Specifically, we used multifocus (MF-PolScope) microscopy to simultaneously capture different Z-positions along with polarized fluorescence information so as to determine if orientation was comparable at all positions in the ring at the same time (Abrahamsson et al., 2015). When the orientation is consistent between Z-slices of the top, middle, and bottom of septin assemblies, we have interpreted this to mean that the prevailing population of septins are “paired filaments” (DeMay et al., 2011a,b).

As previously published from our lab, the septin hourglass of wild-type cells expressing Cdc12-conGFP exhibit GFP dipoles with a net orientation perpendicular to the mother-bud axis, while septin rings exhibit a net orientation parallel to the mother-bud axis (DeMay et al., 2011a,b). The net orientation of GFP dipoles in *bni1*Δ and *bnr1*Δ are comparable to wild-type on average however the anisotropy is lower for both mutants suggesting a slightly less well-ordered structure (Figure 1D, Table 5). We categorized each orientation data point by the state of the ring (broken, aberrant size) but could not see any correlation between grossly misorganized rings and orientations substantially deviating from 90° (Figure 1D). There seems to be little difference between the net orientation in the top, middle, and bottom focal planes in the two formin mutants suggesting pairing or symmetric organization relative to the plasma membrane (Figures 1E,F). In addition, we also estimated the population variance, which is a measure of the difference in average dipole orientation between cells with the same type of structure, and found little difference in each strain over the entire population compared to wild-type cells (Figure 1G). These results suggest that although Bni1 and Bnr1 are required for the proper accumulation and organization of septins in the septin hourglass, they are not required for proper timing of disassembly and reorientation of septins at ring splitting (Figures 1B–E).

**Table 5:** **Average anisotropy for all strains**.

**Strain**	**HG Anisotropy**	**S Anisotropy**
*CDC12*-conGFP	0.25	0.22
*bni1*Δ	0.16	0.18
*bnr1*Δ	0.12	0.18
*bud4*Δ	0.15	0.02
*cla4*Δ	0.13	0.17
*gin4*Δ	0.05	0.17
*rts1*Δ	0.11	0.25
*shs1*Δ	0.03	0.05
*shs1*Δ*C*	0.02	0.06
*CDC3*-GFP (W303)	0.12	0.15
*shs1*Δ*-ps1*	0.01	0.09
*shs1*Δ*-ps2*	0.06	0.01
*shs1*Δ*-ps4*	0.05	0.05

*HG = Hourglass and S = Split*.

### Septins in cells lacking *bud4* have highly misorganized rings after splitting

**Figure 2:**
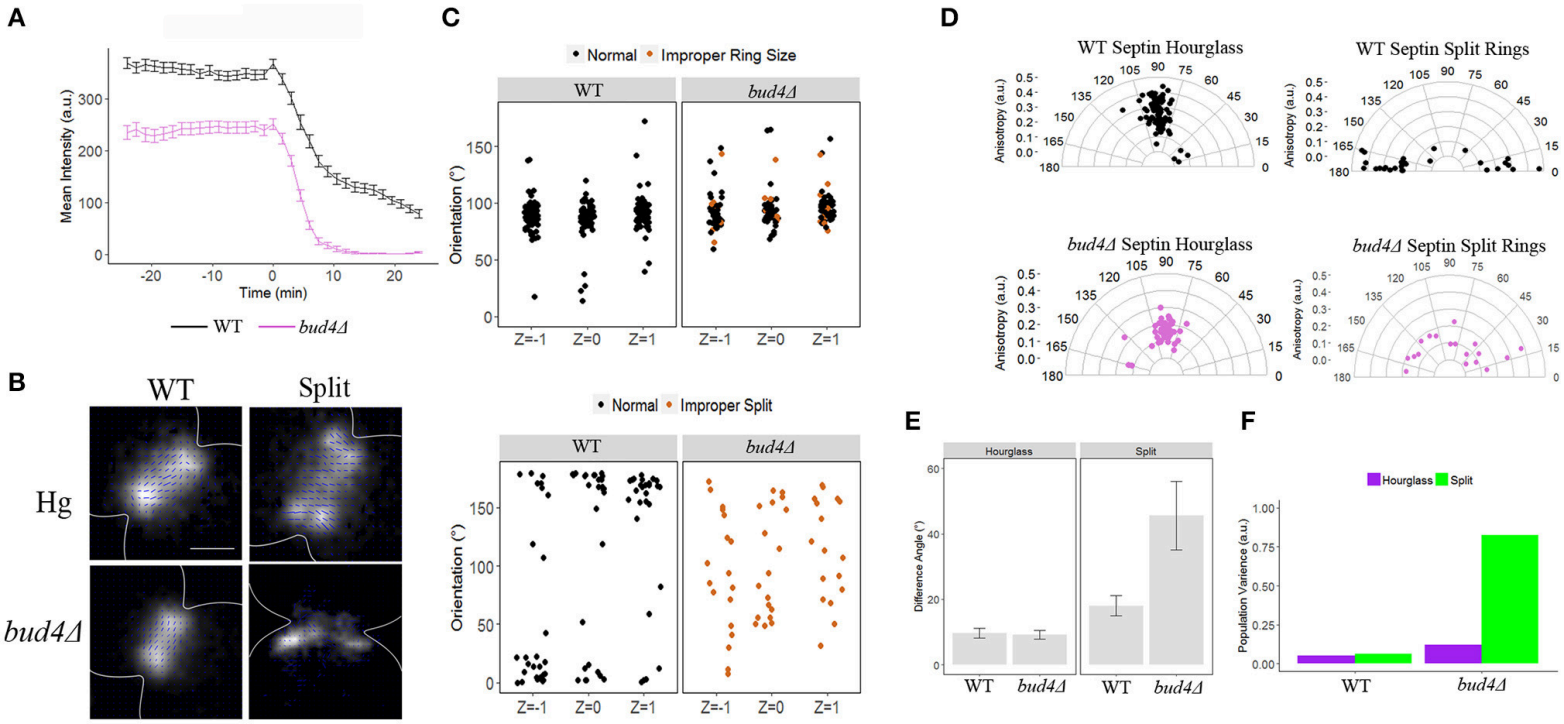
**Analysis of cells lacking Bud4**. **(A)** Septin intensity of GFP-Cdc3 in wild-type (black) and *bud4*Δ (pink) over the course of septin ring splitting (time = 0). Error bars represent standard error for each time point (*N* = 100 cells). **(B)** Representative polarization images of wild-type containing pSc*CDC12*-conGFP, and *bud4*Δ containing pSc*CDC12*-conGFP in the hourglass and split structure. The blue lines represent the calculated dipole orientation and their length is scaled according to anisotropy. Scale bar 0.5 μm. **(C)** Scatter plot of net dipole orientations for individual hourglass and split septin rings in three different focal planes. Color scale indicates visible phenotypes of septin rings [improper ring size (orange), Improper split (orange, lower panel), (hourglass *N* > 50 cells, *N* = 18 cells, respectively)]. **(D)** Polar plots of net dipole orientations for individual hourglass and split septin rings scaled by anisotropy (hourglass *N* > 50 cells, *N* = 18 cells, respectively). **(E)** Difference in net orientation measures between top and bottom focal planes for hourglass and split structures. Error bars denote standard error. **(F)** Population variance in hourglass and split ring structures.

We next examined other septin interacting proteins beginning with Bud4, an anillin-like protein. Bud4 has been shown to associate with septins and mutants do not have two split septin rings making them a key candidate in regulation of septin orientation in this process (Wloka et al., 2011). We looked at septin intensity through time over ring splitting in the *bud4*Δ mutant. We observed septins leave the bud neck in a monophasic manner (2-fold faster than wild-type in phase 1, and 3.5-fold faster than wild-type in phase 2). This rapid disassembly from the bud neck is consistent with previous data that Bud4 is required for stability of split septin rings during and after cytokinesis (Figure 2A, Table 4, Wloka et al., 2011; Eluère et al., 2012; Kang et al., 2013). The ring disassembled asymmetrically with one side much faster than the other but the analysis here was averaged across the whole ring to be consistent with other measurements in the study. Similar to *bnr1*Δ mutants, *bud4*Δ mutants showed a reduced septin accumulation within the hourglass with ~70% the intensity observed in wild-type hourglasses.

Using polarization microscopy, we observed little difference in terms of septin hourglass orientation, a slight decrease in anisotropy, and comparable population variance across all focal planes compared to wild-type (Figures 2B–F, Table 5). Any variability in GFP dipole orientation observed in the *bud4*Δ mutant could not be explained by visible septin defects (Figure 2C). However, when we analyzed the orientation of split rings in the *bud4*Δ we observed a severe decrease in anisotropy, across all focal planes suggesting that Bud4 is also required for proper organization of septin split ring structures (Figures 2C–F, Table 5). This is again consistent with previous data that Bud4 is required for the stability of the septin split rings (Wloka et al., 2011; Eluère et al., 2012; Kang et al., 2013).

### Gin4, but not Cla4 or Rts1, contributes to rate of disassembly at splitting

**Figure 3:**
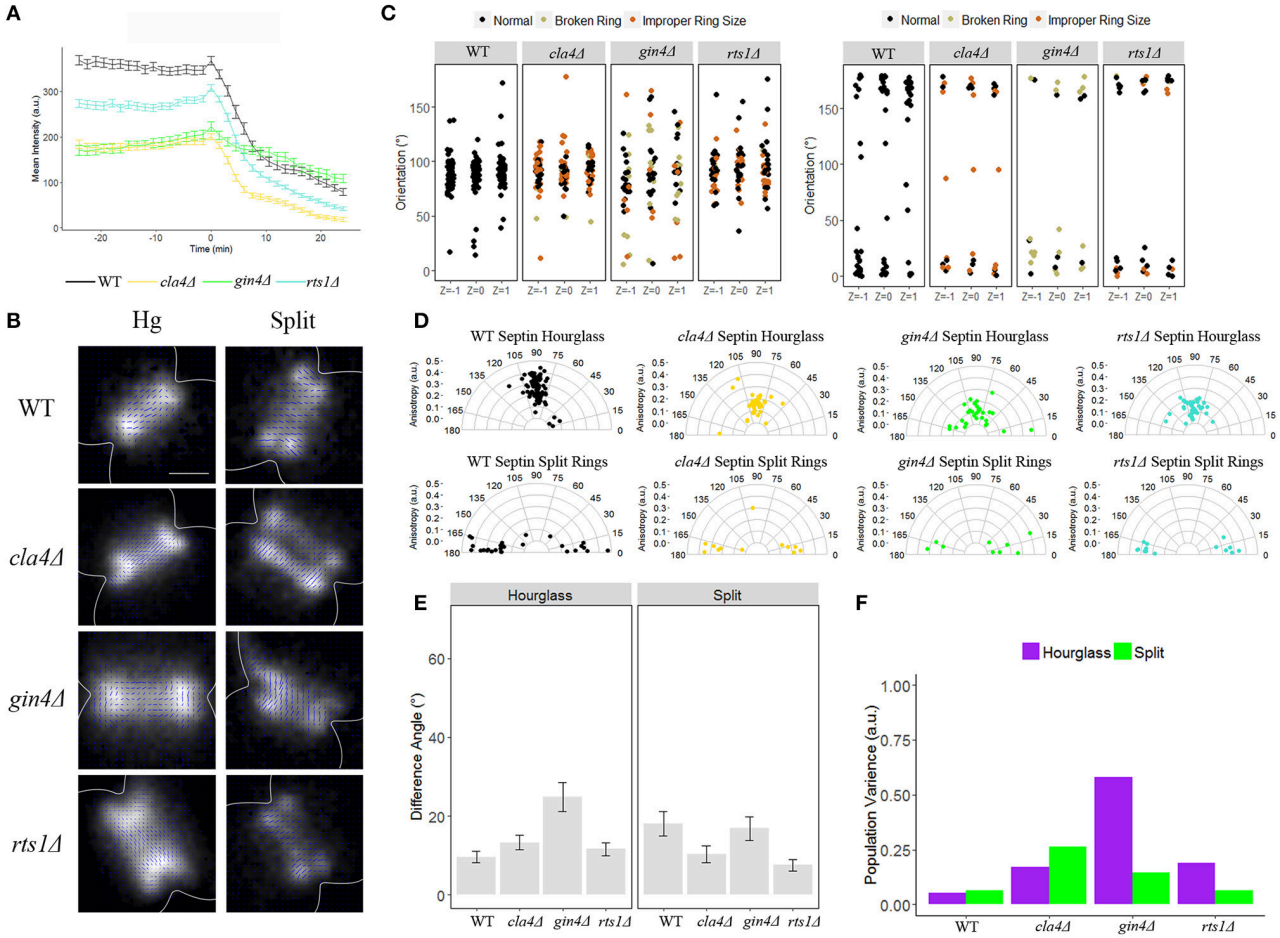
**The kinase Gin4 is required for organization of the septin hourglass, but not the septin split rings. (A)** Septin intensity of GFP-Cdc3 in wild-type (black), *cla4*Δ (yellow), *gin4*Δ (green), and *rts1*Δ (turquoise) over the course of septin ring splitting (time = 0). Error bars represent standard error for each time point (*N* = 100 cells). **(B)** Representative polarization images of wild-type containing pSc*CDC12*-conGFP, *cla4*Δ containing pSc*CDC12*-conGFP, *gin4*Δ containing pSc*CDC12*-conGFP, and *rts1*Δ containing Sc*CDC12*-conGFP in the hourglass and split structure. The blue lines represent the calculated dipole orientation and their length is scaled according to anisotropy. Scale bar 0.5 μm. **(C)** Scatter plot of net dipole orientations for individual hourglass and split septin rings in three different focal planes. Color scale indicates visible phenotypes of septin rings [broken ring (tan), improper ring size (orange), (hourglass *N* > 30 cells, split rings *N* > 9 cells)]. **(D)** Polar plots of net dipole orientations for individual hourglass and split septin rings scaled by anisotropy (hourglass *N* > 30 cells, split rings *N* > 9 cells). **(E)** Difference in net orientation measures between top and bottom focal planes for hourglass and split structures. Error bars denote standard error. **(F)** Population variance in hourglass and split ring structures.

The isotropic transition between the two orthogonal organization states is quite transient, lasting just a few minutes, indicating that the cue to direct disassembly and/or reassembly could be a post-translational modification. Recipients of the modification could be a subpopulation of septins, a septin regulator and/or modifiers of the local membrane composition. We examined two kinases, Cla4 and Gin4, and one phosphatase, Rts1, that have been shown to contribute to septin ring behavior (Longtine et al., 2000; Mortensen et al., 2002; Dobbelaere et al., 2003; Gladfelter et al., 2004; Versele and Thorner, 2004; Roelants et al., 2015). Both *gin4*Δ and *cla4*Δ cells showed a decrease in septin accumulation in the hourglass, both exhibiting ~50% reduction in intensity relative to the wild-type hourglass (Figure 3A, green and gold lines). In addition, we observed that septins do not leave the bud neck in a biphasic manner in the *gin4*Δ mutant (Figure 3A). Instead, septins leave the bud neck at a constant rate that is slower in both phases compared to wild-type cells (~75% the rate of wild-type in phase 1, and ~30% slower than the rate wild-type in phase 2, Table 4). In contrast, in *cla4*Δ cells septins still left the bud neck in a biphasic manner similar to the rate seen in wild-type cells (0.1-fold faster than wild-type in phase 1, and 0.4-fold faster than wild-type in phase 2, Table 4). Notably, Rts1 had little effect on septin accumulation in the hourglass, and no effect on disassembly rate relative to wild-type cells (Figure 3A, Table 4). Thus, Gin4, and to a lesser extent Cla4, are required for the sharp decrease in septin abundance at the neck that accompanies ring splitting.

We next used polarization microscopy to measure the organization of the septin hourglass and split rings in *cla4*Δ, *gin4*Δ, and *rts1*Δ mutant strains (Figures 3B–D). In *cla4*Δ and *rts1*Δ cells the spread of orientations for the GFP-dipole in the hourglass increased (thus the population variance increased), but the net orientation remained ~90° with some decrease in anisotropy (Figures 3C,D,F, Table 5). In both *cla4*Δ and *rts1*Δ strains the split structure was not appreciably different from wildtype (Figures 3B–D). Unlike *cla4*Δ and *rts1*Δ, organization within the septin hourglass in the *gin4*Δ mutant was highly disorganized, while the split structure maintained its ordered, net orientation of ~0/180° (Figures 3B–D). This data indicates that Gin4 is required for ordered septin hourglass organization and efficient disassembly but not the split ring organization. As with *bni1*Δ, *bnr1*Δ, and *bud4*Δ, little difference between the net orientation in the top, middle, and bottom focal planes was observed, and the increase in variability in GFP-dipole orientations within the population could not be correlated with visible septin higher-order structure defects because morphologically aberrant and normal appearing rings had a similar spread of orientations (Figures 3C,E). These data are consistent with a well-appreciated role for Gin4 in establishing the organization of septins but also indicates it is involved in executing the switch-like disassembly process. However, the septins that are recruited/retained after ring splitting do not require Gin4 for normal orientation or order.

### Shs1 contributes to organization of hourglass, split rings and timing of splitting

It is possible that phosphorylation on a subset of septin rod complexes could generate the two distinct pools of septins at cytokinesis that are either in a state of disassembly or stay assembled at the membrane. As Shs1 has the most evidence of phosphorylation, we examined septin organization in cells lacking Shs1 (Mortensen et al., 2002; Egelhofer et al., 2008).

**Figure 4:**
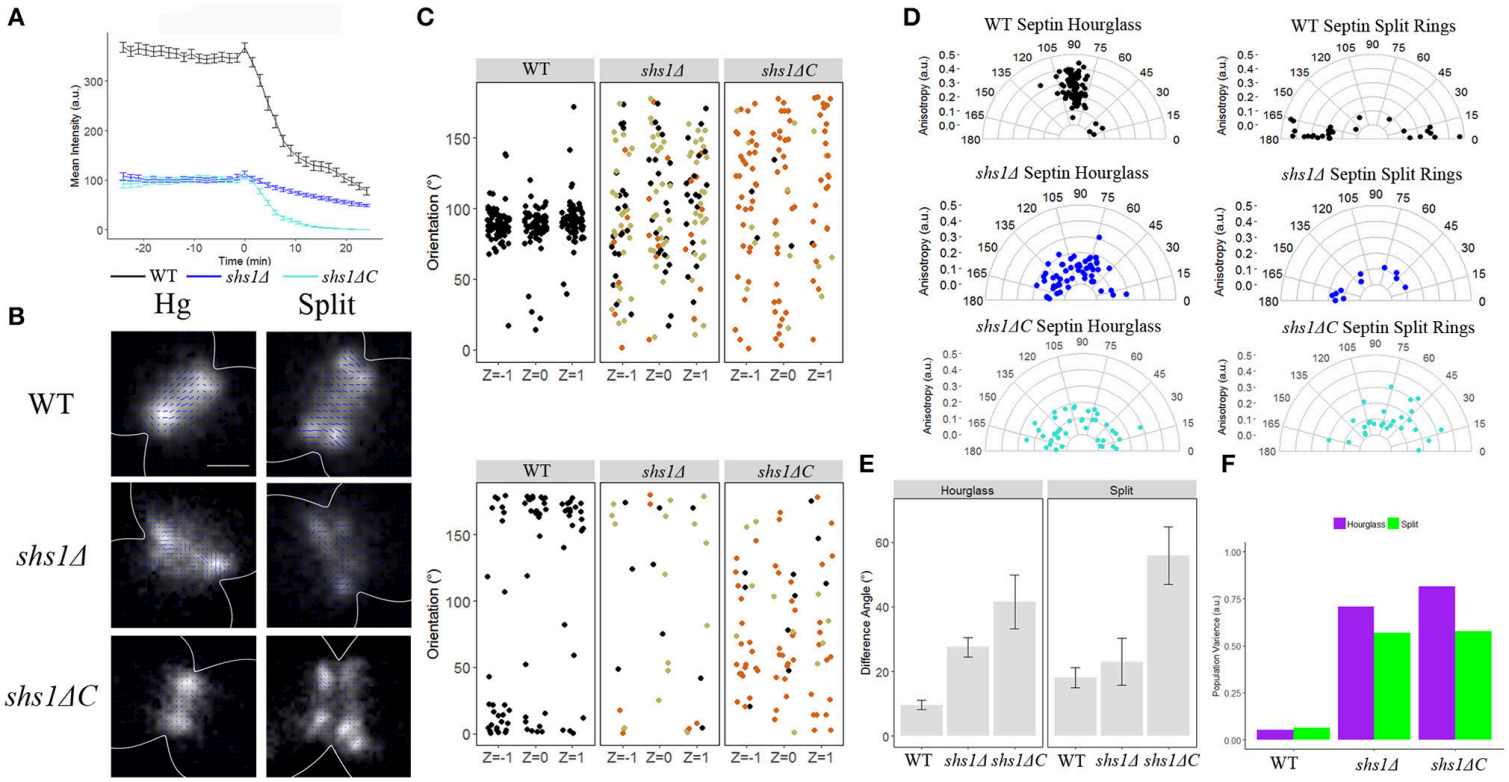
**The septin Shs1 is required for the proper organization of all septin higher order structures. (A)** Septin intensity of GFP-Cdc3 in wild-type (black) and *shs1*Δ (blue) over the course of septin ring splitting (time = 0). Error bars represent standard error for each time point (*N* = 100 cells). **(B)** Representative polarization images of wild-type containing pSc*CDC12*-conGFP, *shs1*Δ containing pSc*CDC12*-conGFP, and *shs1*Δ*C* containing pSc*CDC12*-conGFP in the hourglass and split structure. The blue lines represent the calculated dipole orientation and their length is scaled according to anisotropy. Scale bar 0.5 μm. **(C)** Scatter plot of net dipole orientations for individual hourglass and split septin rings in three different focal planes. Color scale indicates visible phenotypes of septin rings [broken ring (tan), improper ring size (orange), (hourglass *N* > 55 cells, split rings *N* > 10 cells)]. **(D)** Polar plots of net dipole orientations for individual hourglass and split septin rings scaled by anisotropy (hourglass *N* > 55 cells, split rings *N* > 10 cells). **(E)** Difference in net orientation measures between top and bottom focal planes for hourglass and split structures. Error bars denote standard error. **(F)** Population variance in hourglass and split ring structures.

We first looked at septin intensity over ring splitting in a *shs1*Δ mutant strain. We observed a defect in the accumulation of GFP-Cdc3 to the septin hourglass (~70% reduction compared to wild-type intensity), and similar to what we observed in *gin4*Δ mutant cells, a monophasic disassembly rate of septins from the bud neck slower than either observed phase in wild-type cells (0.6-fold slower than wild-type phase 1, and 0.3-fold slower than wild-type phase 2, Table 4, Figure 4A) When observed by polarization microscopy, there was a dramatic increase in the disorganization of both the hourglass and split ring structures in the *shs1*Δ mutant strain, consistent with what has been seen for *shs1*Δ mutants by cryo-EM tomography and platinum replica EM (Figures 4B–D, Bertin et al., 2012; Ong et al., 2014). Septin disorganization was reflected in a wide spread of GFP-dipole orientations and thus an increased variance within the population, and a decrease in anisotropy (Figures 4D,F, Table 5). As with the previous mutant strains we analyzed, the difference in spread of angles was not associated with visible defects in septin structures, and no obvious difference was observed in GFP-dipole net orientation between the top, middle, and bottom focal planes (Figures 4C,E).

It is clear that complete loss of Shs1 leads to gross abnormalities in the construction and organization of septin structures throughout the cell cycle making it difficult to disentangle assembly defects from a role of Shs1 in the splitting and reorganization process. We hypothesized the highly phosphorylated C-terminus of Shs1 could be contributing to septin organization. We therefore, examined cells carrying *shs1*Δ*C*, where the C-terminus of Shs1 is eliminated. *shs1*Δ*C* cells showed a similar decrease in septin accumulation in the hourglass as the *shs1*Δ null strains, but interestingly still showed biphasic disassembly of septins over the course of splitting (Figure 4A, Table 4). When septin organization was assessed using polarization microscopy, both hourglasses and split rings in *shs1*Δ*C* were disorganized, similar to *shs1*Δ (Figure 4, Table 5). Taken together these data suggest the C-terminus of Shs1 is not important for the biphasic exit of septins from the mother-but neck but is necessary for proper organization of septin higher order structures throughout the cell cycle. What aspect of the Shs1 C-terminus makes it important for septin organization but not septin disassembly?

**Figure 5:**
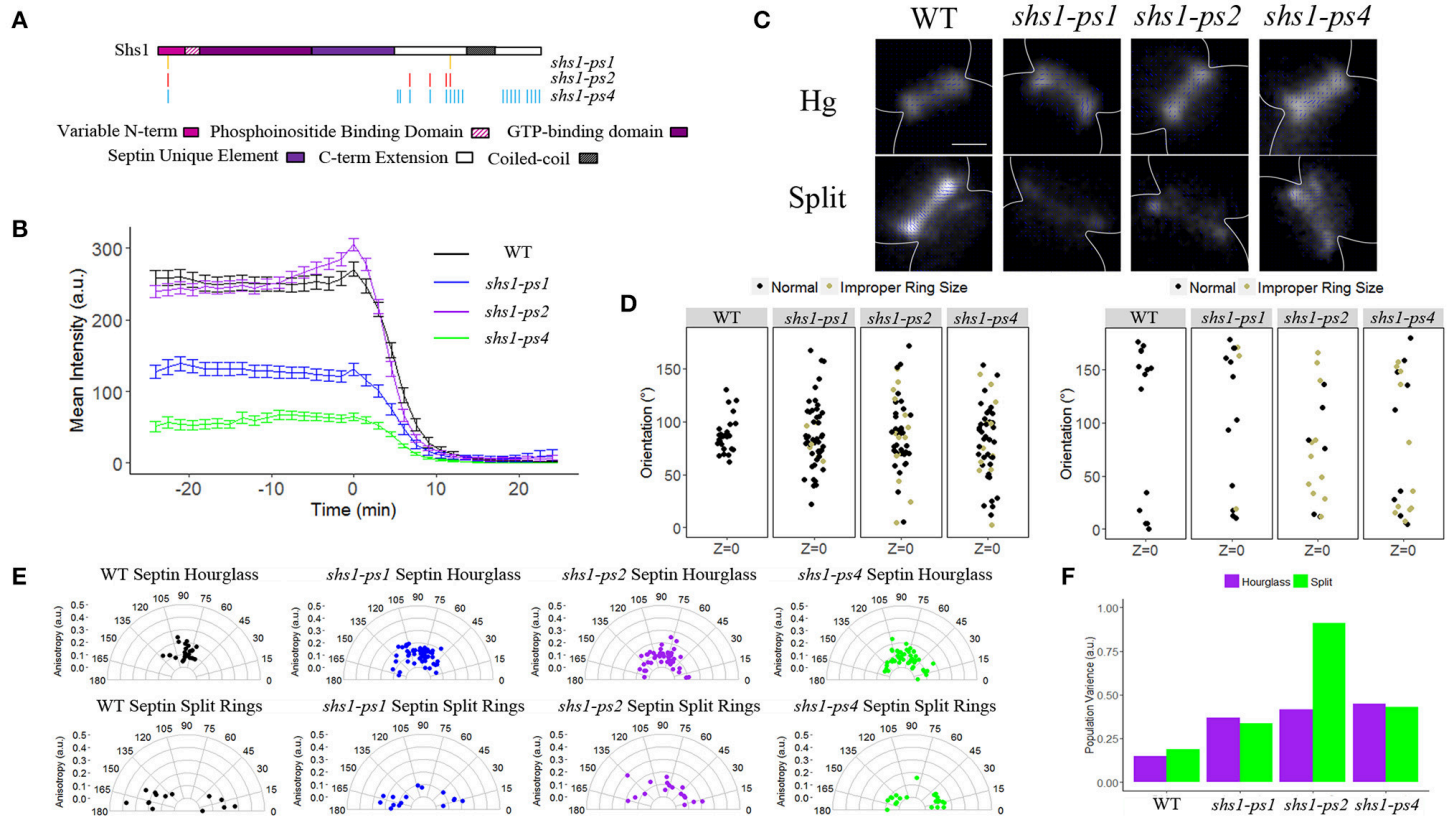
**Shs1 phosphorylation modulates septin abundance, splitting dynamics, and organization. (A)** Schematic of Shs1 protein domains with mutated phosphorylation sites highlighted for each shs1-phosphomutant (adapted from Egelhofer et al., 2008). **(B)** Septin intensity of GFP-Cdc3 in W303 background (black), *shs1-ps1* (blue), *shs1-ps2* (purple), and *shs1-ps4* (green) over the course of septin ring splitting (time = 0). Error bars represent standard error for each time point (*N* = 100 cells). **(C)** Representative polarization images of W303 background containing Sc*CDC12*-conGFP, and *shs1-*phosphomutants containing Sc*CDC12*-conGFP in the hourglass and split structure. The blue lines represent the calculated dipole orientation and their length is scaled according to anisotropy. Scale bar 0.5 μm. **(D)** Scatter plot of net dipole orientations for individual hourglass and split septin rings in three different focal planes. Color scale indicates visible phenotypes of septin rings [broken ring (tan), improper ring size (orange), (hourglass *N* > 26 cells, split rings *N* > 14 cells)]. **(E)** Polar plots of net dipole orientations for individual hourglass and split septin rings scaled by anisotropy (hourglass *N* > 26 cells, split rings *N* > 14 cells]. **(F)** Population variance in hourglass and split ring structures.

We next analyzed the organization of septin higher-order structures in mutants of Shs1 that have altered phosphorylation sites (Egelhofer et al., 2008). Several *shs1* phosphorylation defective mutants were evaluated that had been previously generated based on results from SILAC mass spectrometry experiments in the Kellogg lab. These were ps1 (Pho85 sites), ps2 (CDK sites), and ps4 (Pho85/CDK sites) (Figure 5A; Egelhofer et al., 2008). Septin disassembly rates were notably faster in the wild-type strain background of these mutant alleles, which could be due to background mutations such as in *BUD4*, which is consistent with the observed asymmetric splitting also observed in certain *bud4*Δ strains (Voth et al., 2005). The *shs1* ps mutants showed strikingly distinct assembly and disassembly defects amongst each other. *shs1-ps1* and *shs1-ps4* had substantially diminished levels of septins in assembled hourglasses, ~50 and 80% reduction in intensity relative to wild-type, respectively (Figure 5B). In contrast, *shs1-ps2* cells had similar septin levels at the hourglass compared to wild-type as gauged by GFP-Cdc3 intensity. Interestingly, none of the *shs1* ps mutants had a different rate of disassembly from wild-type (Table 4). This is potentially a result of the strain background interfering with any differences in rates relative to one another; however, these data still suggest that phosphorylation may be able to tune the affinity of septins for membrane at the neck or septin-septin interactions for the assembly of the hourglass.

All the *shs1* phosphorylation-deficient mutants were comparably highly disorganized and showed a wide distribution of orientations and cell-to-cell variability in both hourglass and asymmetric split ring structures (Figures 5C–F, Table 5). The impact of these mutations is comparable to a complete null mutant of *SHS1*. These data attest to the key importance of Shs1 and likely its regulation by phosphorylation in modulating the abundance and organization of septin higher-order structures but do not support a major role of these modifications in the splitting process.

## Discussion

What could specify the bifurcation of behavior within a population of septins such that disassembly and reassembly/retention processes basically coexist in time and space? Our goal was to identify features of septins themselves and regulators that might be responsible for this transition in filament organization. One hypothesis we tested was that the septin Shs1 might specify which septins are prone to departure versus reassembly/retention. The difference in abundance of the two terminal septins within the hourglass is consistent with evidence that Shs1 has the ability to cap septin rods, but not polymerize with other Shs1 containing rods (Finnigan et al., 2015). However, we did not see a substantial difference in the rate at which Cdc11 and Shs1 depart the neck indicating that simply different terminal subunits do not specify the subpopulations at splitting. These results do not lead to a simple explanation for specifying the different fates of the hourglass and split ring septin filaments based on differential behavior of Cdc11 and Shs1 during the transition.

We hoped that through careful analysis of splitting kinetics and organization states that we would be able to dissect the molecular control of the transition. We identified several categories of defects in both the kinetics of splitting and septin organization by combining timelapse imaging with polarized fluorescence analysis (Table 6): (1) Decreased abundance in the hourglass (*bni1*Δ*, bnr1*Δ*, bud4*Δ*, gin4*Δ*, cla4*Δ*, rts1*Δ, *shs1*Δ*, shs1*Δ*C*, and *shs1-ps1* and *-ps4*); (2) Increased rate of disassembly (*bud4*Δ); (3) Monophasic rate of disassembly (*gin4*Δ, *shs1*Δ*);* (4) Misorganized hourglass but organized split rings (*gin4*Δ); (5) Organized hourglass but misorganized split rings (*bud4*Δ); (6) Misorganized hourglass and split rings (*shs1*Δ, *shs1*Δ*C shs1-ps1,2 and 4)*; and (7) correctly oriented but less ordered (low anisotropy) assemblies (*bni1*Δ*, bnr1*Δ*, cla4*Δ*, rts1*Δ). Of note, many regulators (Class1), including those influencing F-actin have an appreciable impact of the abundance of septins in the hourglass structure. It is unclear whether this is due to some altered membrane composition in these mutants or direct impacts on septins that may lower their affinity for the membrane.

**Table 6:** **Results summary**.

**Defect category**	**Mutant(s)**
Decreased abundance in the hourglass	*bni1Δ, bnr1Δ, bud4Δ, gin4Δ, cla4Δ, rts1Δ, shs1Δ, shs1ΔC* and *shs1-ps1* and *-ps4*
Increased rate of disassembly	*bud4*Δ
Monophasic rate of disassembly	*gin4Δ, shs1*Δ
Misorganized hourglass but organized split rings	*gin4*Δ
Oraganized hourglass but misorganized split rings	*bud4*Δ
Misorganized hourglass and split rings	*shs1Δ, shs1ΔC, shs1-PS*
Correctly oriented but less ordered (low anisotropy) assemblies	*bni1Δ, bnr1Δ, cla4Δ, rts1*Δ

In this study, we had aimed to identify mutants that would have specific defects in reorientation. However, the only cases where we see definite defects in reorientation is in scenarios where the ring fails to split or the split rings are unstable such as what happens in cells lacking Bud4. A clear challenge that emerged from this study is that perturbing many of the factors that we suspect might contribute to the process leads to defects in the starting higher-order structure of the septin hourglass. In many cases these defects were not detectable until analysis with polarized light. It is difficult to disentangle the degree to which defects arise due to poor construction as opposed to an inability to respond appropriately to cell cycle triggers of the transition. Future work will need to expand this screen to the vast array of septin regulators and interacting proteins that are known to exist with the hope of still identifying a regulator that might be responsible for this transition without contributing to the organization *per se*. It is likely that this next phase of screening will require identifying partial loss-of-function or separation-of-function alleles because it is probable that whatever responds to or executes the signal for reorientation also contributes to overall septin organization.

Despite the constellation of phenotypes, there are a few key aspects of the data that point to potential mechanisms relevant to the splitting transition. In particular, consistent with previous observations, Bud4 is required for a proper split ring state (Wloka et al., 2011; Eluère et al., 2012; Kang et al., 2013). We were interested to note that the reorientation did not occur in the absence of splitting. This suggests that the reorientation is concomitant with and potentially requires the splitting process. One possible reason for this is that the reorientation also involves reacting to changes in local membrane curvature and composition that are happening at cytokinesis and also contribute to the reassembly process. On a molecular level, the data indicate that either Bud4 is required for receiving the cell cycle cue of splitting and/or is an essential guide or anchor for the reassembly process that creates the split ring structures.

Another clue emerges from the similar behavior of *gin4*Δ and *shs1*Δ which both lose the biphasic disassembly observed in wild-type cells, and thus are slow in going through the transition. This suggests that Gin4 is targeting either Shs1 or is impacting a feature of the transition that Shs1 is required for as well. In the absence of Gin4 activity or Shs1, septins remain associated with the membrane longer. This could be due to Gin4 phosphorylating a subpopulation of septins or other substrates that influence septin affinity for the membrane. We assessed the role of septin phosphorylation (which may or may not be due to Gin4) and see that non-phosphorylatable mutants altered septin levels in the hourglass and monophasic disassembly. Unfortunately, uncertainty about the background of these mutants with regard to the *BUD4* locus makes it challenging to interpret the rate of disassembly in these strains because the disassembly was more rapid than expected in controls. Analysis of an *shs1* mutant that lacks the entire C-terminus was illuminating in that it also has a major defect in the recruitment of septins to the hourglass however it shows a biphasic disassembly that is not slowed compared to wildtype, unlike the *shs1* null. This mutant still retains a phosphorylation site at T6 that is missing in the PS alleles, pointing to this residue as a potentially important site for regulating contact with the membrane for both assembly of the hourglass and release of septins at splitting. Overall, the data point to Gin4 and Shs1 as modulators of the rate of change in septin dynamics and orientation at late stages of the cell cycle. It is likely that this process involves phosphorylation-dependent changes in membrane composition and/or septin-membrane binding that change the affinity of septins for the membrane and possibly change the preference of septins to bind membranes of specific curvatures.

A real power of using polarized fluorescence microscopy is the ability to detect aspects of macromolecular organization not readily apparent in standard total fluorescence measurements and to assess the degree of order in an ensemble of molecules. In our previous work we have seen that no matter how we view the hourglass or rings, the average dipole orientation was the same and we interpreted this to mean that a majority of septins are symmetric relative to the membrane supporting paired filaments. Our motivation for using the MF-PolScope was to be able to simultaneously capture multiple focal planes in the panel of mutants in hopes of detecting defects in pairing that might inform the mechanisms of reorientation. To our surprise, we could see no evidence of systematic differences in orientation that were dependent on focal plane suggesting that pairing may not be impacted in these diverse mutant backgrounds. In summary, this work set out to analyze the requirements for septin rearrangement at cytokinesis in budding yeast. The findings point to a function for Shs1, Gin4, and Bud4 in this process but there is clearly much more work to be done to understand how the dramatic cytoskeletal reorientation occurs in this small window of time and space.

## Author contributions

MM performed experiments, assembled figures, analyzed data and wrote manuscript, MJ performed experiments and analyzed data, SM and AV assisted in data analysis, RO contributed instrumentation and expertise in analysis, AG designed the study and wrote the paper.

## Conflict of interest statement

The authors declare that the research was conducted in the absence of any commercial or financial relationships that could be construed as a potential conflict of interest.
